# Genetic and physiological requirements for high-level sesquiterpene-production in tomato glandular trichomes

**DOI:** 10.3389/fpls.2023.1139274

**Published:** 2023-03-03

**Authors:** Ruy W. J. Kortbeek, Marc D. Galland, Aleksandra Muras, Rodrigo Therezan, Sofia Maia, Michel A. Haring, Robert C. Schuurink, Petra M. Bleeker

**Affiliations:** Green Life Science Research Cluster, Swammerdam Institute for Life Sciences, University of Amsterdam, Amsterdam, Netherlands

**Keywords:** tomato, glandular trichome, sesquiterpenes, MEP pathway, metabolic activity, *Solanum habrochaites*, specialised metabolism

## Abstract

Type-VI glandular trichomes of wild tomato *Solanum habrochaites* PI127826 produce high levels of the sesquiterpene 7-epizingiberene and its derivatives, making the plant repellent and toxic to several pest insects and pathogens. How wild tomato trichomes achieve such high terpene production is still largely unknown. Here we show that a cross (F1) with a cultivated tomato produced only minute levels of 7-epizingiberene. In the F2-progeny, selected for the presence of the 7-epizingiberene biosynthesis genes, only three percent produced comparable amounts the wild parent, indicating this trait is recessive and multigenic. Moreover, trichome density alone did not explain the total levels of terpene levels found on the leaves. We selected F2 plants with the “high-production active-trichome phenotype” of PI127826, having trichomes producing about 150 times higher levels of terpenes than F2 individuals that displayed a “low-production lazy-trichome phenotype”. Terpene quantities in trichomes of these F2 plants correlated with the volume of the storage cavity and shape of the gland. We found that trichome morphology is not a predetermined characteristic, but cavity volume rather depended on gland-cell metabolic activity. Inhibitor assays showed that the plastidial-precursor pathway (MEP) is fundamental for high-level production of both cytosolic as well as plastid-derived terpenes in tomato trichomes. Additionally, gene expression profiles of isolated secretory cells showed that key enzymes in the MEP pathway were higher expressed in active trichomes. We conclude that the MEP pathway is the primary precursor-supply route in wild tomato type-VI trichomes and that the high-production phenotype of the wild tomato trichome is indeed a multigenic trait.

## Introduction

Trichomes are hair-like structures present on the aerial parts of approximately 30% of all land plants and they constitute an important part of the plant’s defence system against herbivorous insects. Non-glandular trichomes form a physical barrier e.g. by impeding movement over the plant’s surface, perturbating feeding behaviour or disruption of the insect digestive system (Kariyat et al., 2017; Andama et al., 2020). Glandular trichomes can produce, store and/or secrete a large number of volatile-and non-volatile specialised metabolites. These metabolites act as defence compounds by being repellent, causing toxicity, interfere with insect development, entrap them, or attract predatory insects (Glas et al., 2012; Kortbeek et al., 2019; Schuurink and Tissier, 2020).

The tomato genus displays seven different trichome types; non-glandular type II, III, V and glandular types I, IV, VI and VII (Luckwill, 1943). The presence and distribution of these depend on the tomato species, accession and tissue, and can be influenced by environmental conditions or stresses (Vendemiatti et al., 2017; Chen et al., 2018; Escobar-Bravo et al., 2018). Some accessions of the wild tomato *Solanum habrochaites*, including PI127826 and LA1777, are known to produce high amounts of specific sesquiterpenoids in their type-VI trichomes making them resistant against multiple insects and pathogens (Fridman et al., 2005; Sallaud et al., 2009; Schilmiller et al., 2009; Bleeker et al., 2011a; Akhtar et al., 2013; Nakashima et al., 2016). This trichome type consists of four secretory cells on top of a multicellular stalk and they can be found on the green parts of most tomato species, including cultivated tomato (*S. lycopersicum*; Glas et al., 2012).

In both *S. lycopersicum* and *S. habrochaites*, sesquiterpenes are synthesised in the cytosol from acetyl-CoA *via* the mevalonate (MVA) pathway (Hemmerlin et al., 2012) with some enzymes localised to the peroxisomes. Here, acetyl-CoA is first metabolised to the common C_5_ isoprenoid precursors isopentenyl diphosphate (IPP) and its isomer dimethylallyl diphosphate (DMAPP). Next, prenyltransferase farnesyl diphosphate synthase (FPS) catalyses the head-to-tail condensation of IPP/DMAPP to form the C_15_ precursor farnesyl diphosphate (FPP). In cultivated tomato, FPP is mainly utilised by the sesquiterpene synthase *Sl*TPS12 to form α-humulene and β-caryophyllene while *S. habrochaites* additionally produces a mix of germacrenes *via Sh*TPS9 (Schilmiller et al., 2010; Bleeker et al., 2011b). Both species also generate IPP/DMAPP from pyruvate and glyceraldehyde 3-phosphate (G3P) *via* the plastidial 2-C-methyl-D-erythritol 4-phosphate pathway (MEP). In trichomes of cultivated tomato, IPP/DMAPP are condensated by a *cis*-prenyltransferase, neryl-diphosphate synthase 1 (NDPS1), to generate the C_10_ monoterpene-precursor neryl-diphosphate (NPP; Schilmiller et al., 2009). NPP is utilised predominantly by phellandrene synthase 1 (PHS1, also referred to as TPS20) generating a mix of monoterpenes (Schilmiller et al., 2009). In the plastids of *S. habrochaites* accessions the *cis*-allelic variant of NDPS1, *Z*-isoprenyl pyrophosphate synthase (zFPS), condensates IPP/DMAPP to the C_15_ sesquiterpene precursor *Z*,*Z*-farnesyl pyrophosphate (zFPP; Sallaud et al., 2009; Kang et al., 2014) which is utilised by *S*. *habrochaites*-specific sesquiterpene terpene synthases. In PI127826 the 7-epizingiberene synthase (ZIS) generates 7-epizingiberene (7epiZ) and *R*-curcumene (Bleeker et al., 2012) while in LA1777 the santale/bergamotene synthase (SBS) forms a mix of santalenes and bergamotenes (Sallaud et al., 2009). In some accessions, these sesquiterpenes are further oxidised by which they gain toxicity to insects and/or pathogens (Frelichowski and Juvik, 2001; Zabel et al., 2021).

In type-VI trichomes, terpenes are stored inside an intercellular storage-cavity, located central to the four glandular cells (Tissier et al., 2017). Wild tomato genotypes like *S. habrochaites* often have larger storage cavities compared to trichomes of cultivated tomatoes, and this size difference seems to be positively correlated to quantity of specialised metabolites inside the trichome (Bergau et al., 2015). Furthermore, type-VI trichomes can differ in morphology. The shape of the glandular heads on cultivated tomato are often described as “lobed” or “clover shaped”, referring to the four secretory cells which are distinctly visible. In contrast, the glandular heads of some *S*. *habrochaites* accessions, e.g. LA1777 or PI126449, are referred to as “globular” or “spherical”, in which the individual secretory cells are not distinguishable (Ben-Israel et al., 2009; Bergau et al., 2015).

Despite the differences in biochemistry and morphology between *S*. *lycopersicum* and *S*. *habrochaites* trichomes, it is possible to transfer terpenoid production from a wild species to a cultivar as both species have active MVA and MEP pathways providing the universal precursor IPP/DMAPP. Indeed, we previously showed that that transgenic cultivated tomato can produce sesquiterpene 7epiZ after introducing two genes only; i.e. *zFPS* and *ZIS* from PI127826 (Bleeker et al., 2012). Similarly, we showed that santalenes/bergamotenes can be produced by cultivated tomato trichomes with the introgression of the *zFPS* and *SBS* locus (*Sst2*) from LA1777 to a *S. lycopersicum* cultivar (cv), MicroTom (Therezan et al., 2021). However, in all these cases terpene quantities remained relatively low compared to the wild species. The transgenic *zFPS/ZIS* tomato plants, for example, produced ~200 times less 7epiZ compared to PI127826 (Bleeker et al., 2012). This implies that besides the prenyl-transferases and terpene synthases, additional factors are required to achieve high-level production of these terpenes. The previously reported 7epiZ-oxidase (*Sh*ZO) of *S*. *habrochaites* PI127826 is responsible for the oxidation of 7epiZ to 9-hydroxy-zingiberene (9HZ) and 9-hydroxy-10,11-epoxyzingiberene (9H10epoZ), metabolites that are toxic to whiteflies and several microbes in a dose-dependent manner (Zabel et al., 2021). When produced in sufficient quantities, these metabolites could protect the pest-sensitive cultivated tomato, acting as natural defence compounds.

In this paper, we aimed to study the genetic basis of the high-terpene production and storage capacity of wild tomato type-VI trichomes. We generated an interspecific F2 population of a cultivated tomato and *S. habrochaites* PI127826 and showed that it requires multiple recessive loci for an individual trichome to produce 7epiZ in high concentrations, a trait independent of having a high density of glandular trichomes. We selected individuals with contrasting terpene-production phenotypes while being homozygous for the *S. habrochaites* alleles required for the production of 7epiZ and derivatives (i.e. *zFPS*, *ZIS* and *ShZO*). This material enabled us to conclude the capacity to store high terpene requires multiple recessive loci. The large storage volume, and consequent morphology, of *S. habrochaites* type VI-trichomes is the result of a high-level MEP pathway activity.

## Results

### The genetic background of a cultivated tomato impedes 7-epizingiberene biosynthesis

**Figure 1:**
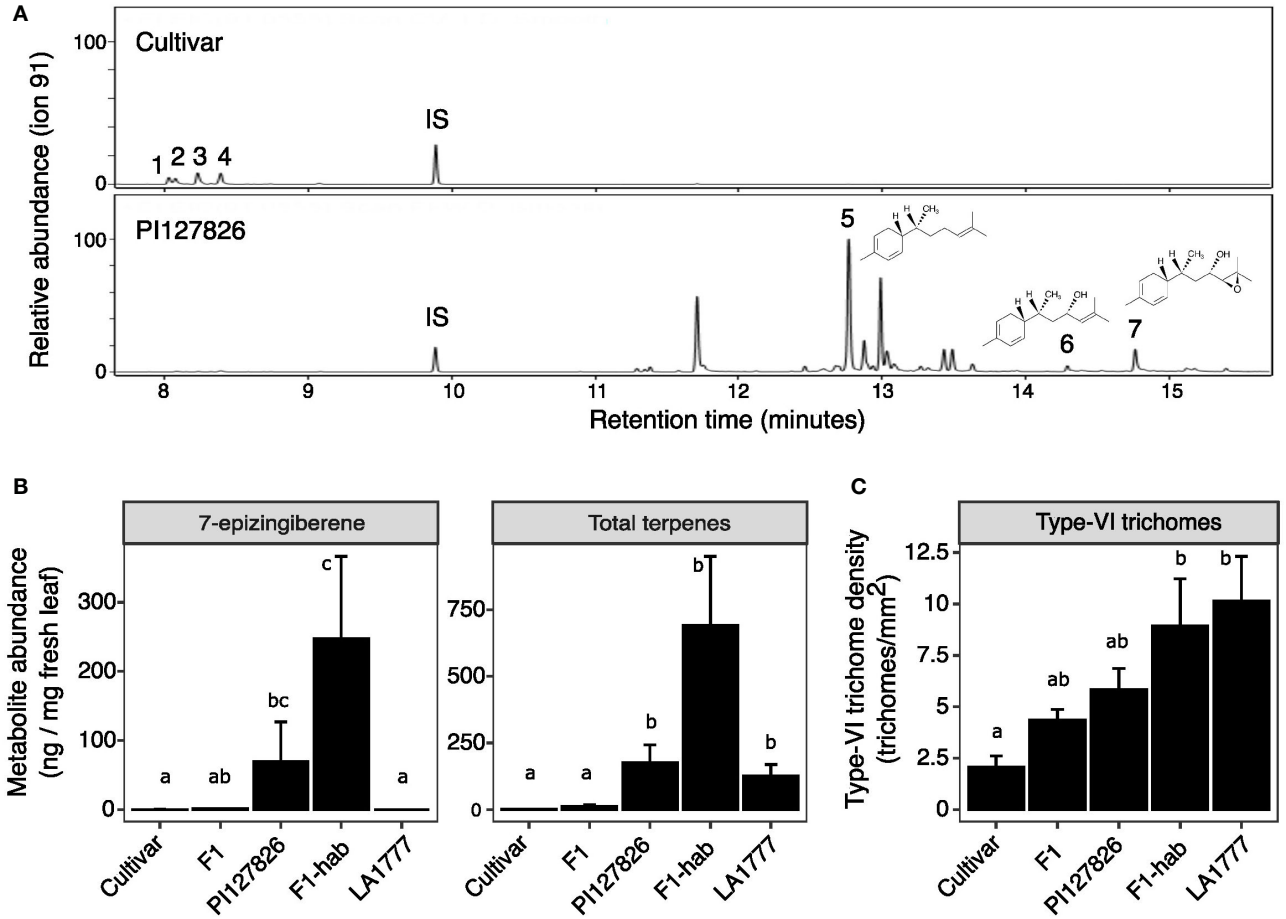
Metabolite levels and trichome density on leaves of tomato hybrids and their parents. **(A)** GC-MS chromatograms of the *S. lycopersicum* cultivar and *S. habrochaites* PI127826. Peaks: (1) 2-carene; (2) α-phellandrene; (3) terpinolene; (4) β-phellandrene/D-limonene; (5) 7-epizingiberene; (6) 9-hydroxy-zingiberene; (7) 9-hydroxy-10,11-epoxyzingiberene; (IS) internal standard. Molecular structures are given for 5, 6 and 7. **(B)** 7-epizingiberene and total mono-and sesquiterpene abundance on the leaves of the cultivar x PI127826 F1 hybrid (F1), the *S. habrochaites* hybrid PI127826 x LA1777 (F1-hab) and their parents. Bars represent mean level of metabolites per mg fresh leaf ± SE (n = 3). **(C)** Type-VI trichomes density on the leaf. Bars represent mean ± SE (n = 3) summed densities over the abaxial and adaxial surface. Statistics were performed after log transformation; letters indicate significant groups (p < 0.05) according to a Tukey HSD *post-hoc* test after ANOVA.

To study the production of 7epiZ and its derivatives 9HZ and 9H10epoZ in the genetic background of a cultivated tomato, the leaf surface of an interspecific F1 cross between PI127826 (male) and a cultivated tomato (female) were analysed for trichome densities and terpene abundance. Leaf washes of PI127826 revealed a total of 176 ng mg^-1^ FW terpenes with 7epiZ as the most abundant terpene (70 ng mg^-1^), along with its derivatives 9HZ (12 ng mg^-1^) and 9H10epoZ (10 ng mg^-1^; **Figure 1A**; **Supplemental Figure S1**). As expected, the quantity of terpenes found in leaf washes of the cultivar were much lower with a total of 1.6 ng mg^-1^ FW, of which monoterpene β-phellandrene was the most abundant (0.5 ng mg^-1^ FW). Surprisingly, the F1 plants also exhibited much lower terpene levels compared to the wild parent. Total terpene levels in the F1 were 12.3 ng mg^-1^ FW, of which 1.1 ng mg^-1^ was 7epiZ, 0.2 ng mg^-1^ 9HZ, 0.8 ng mg^-1^ 9H10epoZ and 2.5 ng mg^-1^ β-phellandrene (**Figure 1A**; **Supplemental Figure S1**). Interestingly, when PI127826 was crossed to *S. habrochaites* accession LA1777, resulting in an *S. habrochaites* F1 (F1-hab), 7epiZ levels of the hybrid exceeded to those of PI127826 (**Figure 1A**). Total terpene content in F1-hab leaf extracts also exceeded those of both parents, mostly through the additional production of bergamotenes and santalenes (**Supplemental Figure S1**).

The quantity of trichome-derived mono-and sesquiterpenes on leaf surface is a result of the total number of the terpene-producing type-VI trichomes multiplied by the production per individual trichome. To explain the differences in terpene levels found, we counted type-VI trichomes on both the adaxial and abaxial leaf surface (**Figure 1C**). Additionally, we recorded acylsugar-producing glandular type-I and IV (taken together as type-I/IV) and non-glandular (NG) trichomes (**Supplemental Figure S2**). Type-VI trichome density of PI127826 appeared to be ~2 times higher compared to the cultivar, whereas the density on the interspecific F1 exhibited an intermediate phenotype (**Figure 1B**). This intermediate phenotype of the F1 was also observed for type-I/IV and NG trichome density. Type-I/IV trichomes were typically highly abundant in PI127826 but absent on leaves of the cultivar. On the contrary, NG trichome density was low on PI127826 and high on the cultivar (**Supplemental Figure S2**). In addition, F1-hab also displayed an intermediate type-VI trichome-density phenotype between both *S. habrochaites* parents PI127826 and LA1777 (**Figure 1C**). The difference in type-VI trichome density did not fully explain the difference in terpene phenotype. Terpenes levels in the F1 were much lower than expected from the inherited genetic dose of the wild parent.

### High-level terpene production comprises a multigenic trait

**Figure 2:**
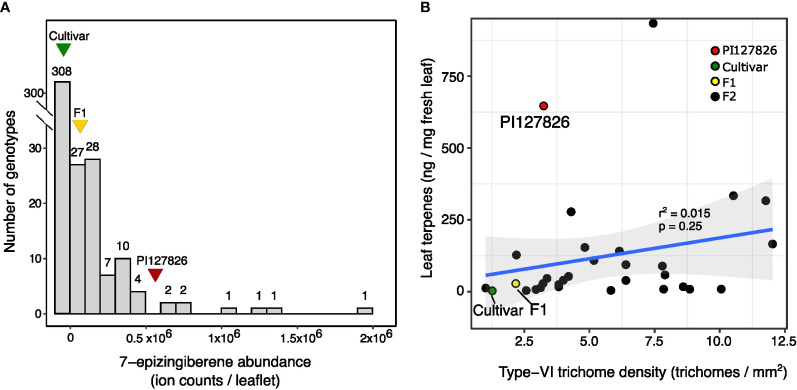
Distribution of 7-epizingberene levels and trichome density of the F2 population. **(A)** Histogram shows the distribution of 7-epizingiberene levels on leaflets of 393 F2-genotypes selected for the presence of *zFPS*, *ZIS*, and *ShZO.* F2 plants are assigned to bins of ion counts of the 7-epizingiberene peak as determined by GC-MS. The width of each bin represents one time the standard deviation of 7-epizingiberene in PI127826 (n =4), with the first bin displaying the number of F2 genotypes with zero ion counts. The number on top of each bar displays the number of F2 genotypes assigned to the particular bin. Triangles indicate the bin with the average 7-epizingiberene levels of the respective parental genotypes (n = 4). **(B)** Scatterplot showing the type-VI trichome density of the sub-population (averaged abaxial/adaxial) versus the total terpene levels (i.e. summed mono-and sesquiterpenes) on the leaves of F2 plants from the sub-population. The blue line indicates the linear correlation plus 95% confidence interval (grey). The correlation coefficient (r^2^) and p-value are displayed on top of the line. Each dot represents measurements of an individual plant.

To investigate the genetics behind the quantitative traits involving terpene production by type-VI trichomes, we created an F2 population of the cultivar and PI127826 by selfing the F1. Next, a subpopulation was selected by screening the F2 progeny for homozygous alleles of *ShzFPS*, *ShZIS* (Solyc08g005640; Solyc08g005680) and *ShZO* (Solyc01g008670) to fix the synthesis of 7epiZ and its derivatives. This subpopulation (n = 392) was screened for mono-and sesquiterpene abundance on the leaf and 7epiZ, being the most abundant terpene, was taken as a proxy. The distribution of 7epiZ levels measured in the population were not normally distributed but instead appeared highly skewed towards low quantities (**Figure 2A**). In fact, only 84 F2 plants had notable levels of 7epiZ, of which 12 displayed parental PI127826 levels or higher. Presuming a Mendelian inheritance, the 12:380 (1:32) segregation of the “high terpene level” phenotype indicates that multiple unlinked loci from the wild parent required. Testing different genetic models, the combination of two recessive and two dominant loci from PI127826 best fitted the observed segregation pattern (**Table 1**). When taking the production of 7epiZ derivatives into account as well, this model again fitted our observations best (**Supplemental Table S1**).

**Table 1:** Comparison of observed 7-epizingiberene segregation to theoretical models.

Gene model	Segregation	χ^2^	p-value
a - b	1: 15	6.80	< 0.01
a - b - C	1: 20	2.50	0.11
a - b - c	1: 63	2.54	0.11
a - b - C - D	1: 27	1.15	0.28
a - b - c - D	1: 84	6.46	< 0.05
a -b -c - d	1: 255	47.35	< 0.01

Gene models consist of recessive (small letters) and dominant (capital letters) loci. The theoretical segregation pattern of the phenotype based on the corresponding gene model is given under Segregation. Models were compared to the observed low:high 7-epizingiberene segregation of 1:32 using the Chi-square test for goodness-of-fit (χ^2^).

To assess the contribution of trichome density to the 7epiZ levels, we counted the type-VI trichomes on leaf discs taken from the F2 plants and the F2 genotypes were grouped into 10 classes according to the summed number of trichomes on both sides (**Supplemental Figure S3A**). Next, we compared the trichome densities to 7epiZ levels using the 84 F2-genotypes with notable 7epiZ levels. Surprisingly, mean 7epiZ levels did not significantly differ between trichome-density classes (ANOVA; p > 0.05; **Supplemental Figure S3B**). Moreover, the 12 F2 genotypes with parental PI127826 7epiZ levels were represented in five of the 10 trichome-density classes, ranging from the class with a low number of trichomes per leaf disc to the class with the highest number of trichomes (**Supplemental Figure S3B**). We selected a subset of F2-plants originating from each trichome-density class (**Supplemental Figure S3C**) and with high and low levels of 7epiZ to investigate the relationship between trichome density and the abundance of terpenes in closer detail. Hereto we quantified and summed the mono- and sesquiterpenes on the leaves and determined the trichome density per mm^2^ of the leaf. This confirmed that that, in this subset of plants, there is no significant linear correlation between the type-VI trichome-density and the quantity of volatile terpenes per mg of fresh leaf (**Figure 2B**; r^2^ = 0.015, p = 0.25).

The results indicate that there is a large variation in volatile production by type-VI trichomes between F2-genotypes which explains why high 7epiZ levels can be found on leaflets with low trichome densities and vice versa. It moreover suggests that trichome density and terpene production by type-VI trichomes segregate independently and should be regarded as genetically separate traits.

### Gland-cell metabolic activity: Active and lazy trichomes

**Figure 3:**
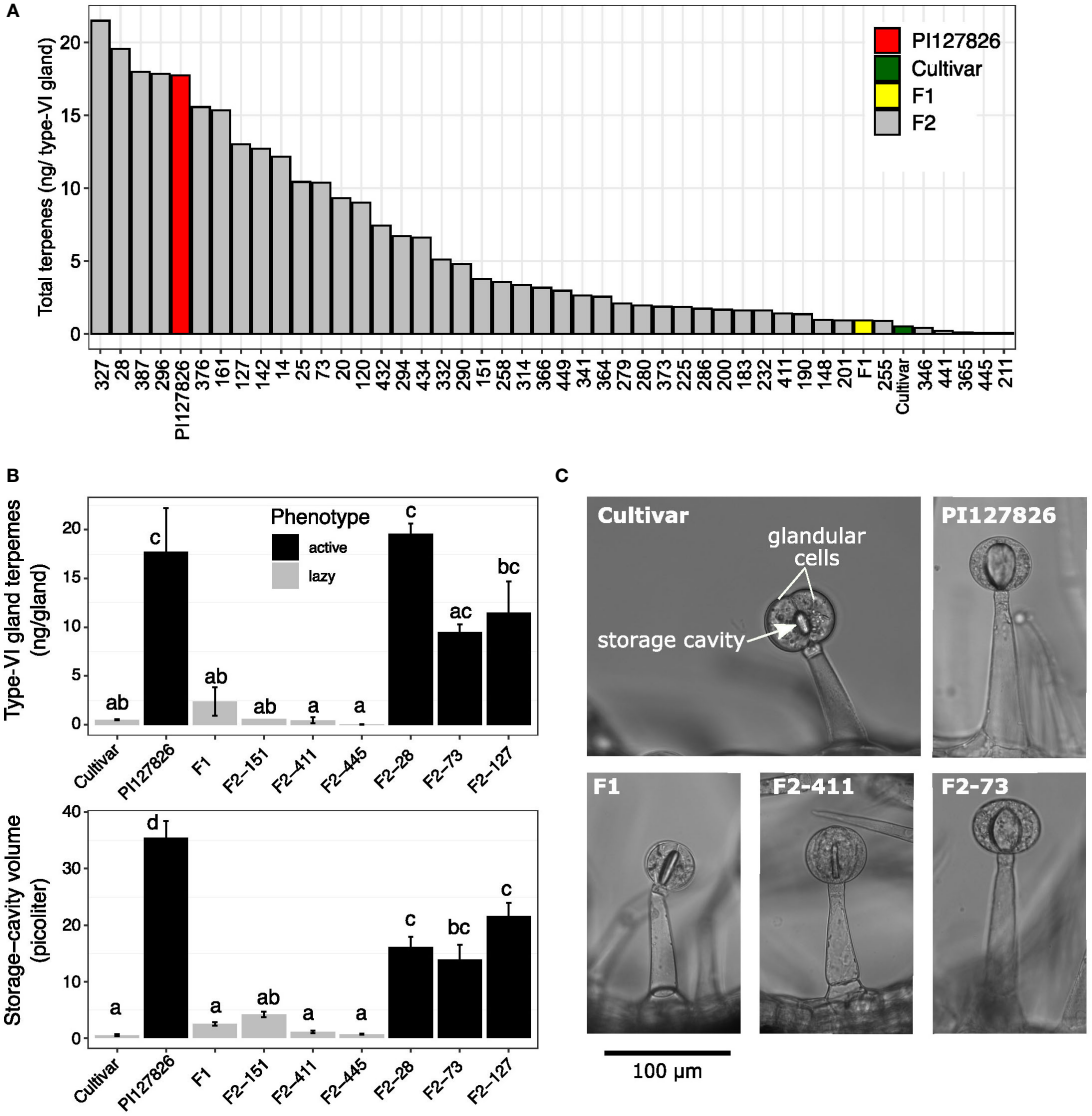
Volatile levels in relation to type-VI trichome densities of the F2 progeny. **(A)** Total amount of mono-and sesquiterpenes per type-VI glandular head of the selected F2-individuals (n = 1) and their parents. **(B)** Representative photographs showing the middle section of type-VI trichomes on the parental genotypes and an active (F2-73) and lazy (F2-411) F2 individuals. Visible are two glandular cells surrounding the central-storage cavity. **(C)** Quantification of the storage-cavity volume (upper panel) of type-VI trichomes (n = 10-20) and total mono-and sesquiterpenes per gland (lower panel) of the selected active (black bars) and lazy (grey bars) F2-genotypes and the parents (n = 3). Letters indicate significant groups (p < 0.05) according to Tukey HSD *post-hoc* test after ANOVA.

To investigate the accumulated quantity of terpenes by type-VI trichomes, the glandular heads (“glands”) were isolated from the subset of F2-plants and analysed by GC-MS. Type-VI glands of the cultivar had an averaged total terpene content of 0.51 ng/gland, mainly consisting of plastidial monoterpenes and to a lesser extend cytosolic sesquiterpenes. Terpene levels of PI127826 type-VI glands were ~35 times higher with 17.7 ng/gland, while levels in F1-glands were ~1.8 times higher (0.9 ng/gland) compared to the cultivar. A broad range of terpene levels was found in glands of the selected F2 progeny ranging from 0.05 ng/gland up to 21.5 ng/gland (**Figure 3A**).

From the available material, we selected six F2-individuals based on their extreme terpene levels in the type-VI glands. Three F2-genotypes exhibited relatively high terpene quantities per gland, hence we refer to them as having “active” trichomes. Another three F2-genotypes were selected for their low levels of terpenes per gland, referred to as having a “lazy” trichome phenotype (**Figure 3B**, upper panel). Light microscopy allowed visualisation of the internal storage cavity of their type-VI trichomes making the different phenotypes clearly visible (**Figure 3C**) and made it possible to calculate the volume of the storage cavities. The storage-cavity volume on the leaves of lazy F2 genotypes ranged from 0.7 picolitre (F2-455) to 4 picolitre (F2-151). Storage-cavity volumes of the active F2 plants ranged from 14 picolitre (F2-28) up to 22 picolitre (F2-127). The cavity volumes of the glands (**Figure 3B**, lower panel) largely mirrored their terpene content, where it appears that larger cavity volumes corresponded to larger quantities of terpenes.

It is furthermore worthwhile to note that we did not observe large variations in gland volume of type-VI trichomes within a genotype; a single F2 individual had either all active or all lazy trichomes, but never a mixture. Additionally, the cavity volume seems to be correlated to the shape of the glands. Active genotypes with large cavities had smooth and spherical-shaped glands while on lazy genotypes the contours of the individual cells became visible and therefore the glands appeared to be more lobed (**Supplemental Figure S4**).

### Inhibition of terpene metabolic flux

**Figure 4:**
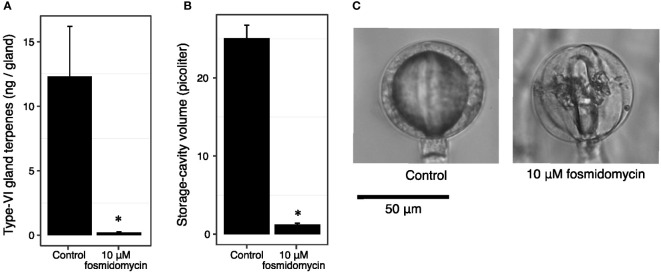
Fosmidomycin treatment of PI127826 cuttings reduces terpene content and storage cavity volume of type-VI trichomes. Cuttings of PI127826 (n = 3) were grown for 14 days in hydroponic solution supplied with or without 10 μM fosmidomycin. **(A)** Quantification of total mono-and sesquiterpene terpene levels in type-VI glands. **(B)** Volume of the storage cavity. **(C)** Representative photographs of a type-VI head displaying the central storage cavity after 14 days under control conditions or with fosmidomycin treatment. Significant differences are annotated with an asterisk: T-test p < 0.01.

To investigate whether cavity volume is a fixed trait that facilitates terpene accumulation, or rather is a consequence of terpenoid accumulation, we inhibited the flux through the plastidial precursor pathway. Cuttings of PI127826 were treated with 10 µM fosmidomycin, inhibiting 1-deoxy-D-xylulose 5-phosphate reductoisomerase (DXR) activity (Zeidler et al., 1998). After 14 days of treatment, the type-VI trichome head-cells from newly developed leaves were analysed for terpene content and storage-cavity volume. In control plants, terpene levels in PI127826 glands measured 12.3 ng/gland, which was reduced with 98% to 0.21 ng/gland in fosmidomycin-treated cuttings (**Figure 4A**). Inhibition of the MEP-pathway did not only affect the accumulation of terpenes produced in the plastid, but also sesquiterpenes produced by cytosol-localised terpene synthases (**Supplemental Figure. S5**). In addition, the treatment reduced the cavity volume of the glands with 95% from 25.0 picolitre in control plants to 1.2 picolitre in treated cuttings (**Figures 4B, C**). The change in cavity volume was accompanied by a difference in physical appearance of the glands. Instead of having smooth and spherical-shaped glands, newly formed glands of fosmidomycin-treated cuttings appeared more lobed (**Figure 4C**) as were the glands of the above mentioned lazy F2-genotypes. Interestingly, type-VI trichomes on leaves that were already present on the cuttings prior to the treatment were much less affected in cavity size and shape; fosmidomycin reduced the cavities on these trichomes with only 16% (**Supplemental Figure S6**).

Following up, we treated both active and lazy F2-genotypes with selective inhibitors to examine if the MEP and the MVA pathway contribution differently to terpene accumulation depending on the genotype. Cuttings of PI127826 and F2-73 (“active”), the cultivar and F2-411 (“lazy”) were treated with 10 µM fosmidomycin to block the MEP pathway, or 10 µM mevastatin to block the MVA pathway by inhibition of HMG-CoA Reductase (HMGR). After 14 days of treatment, type-VI trichome-heads were isolated from newly formed leaves and their terpene content was analysed.

**Figure 5:**
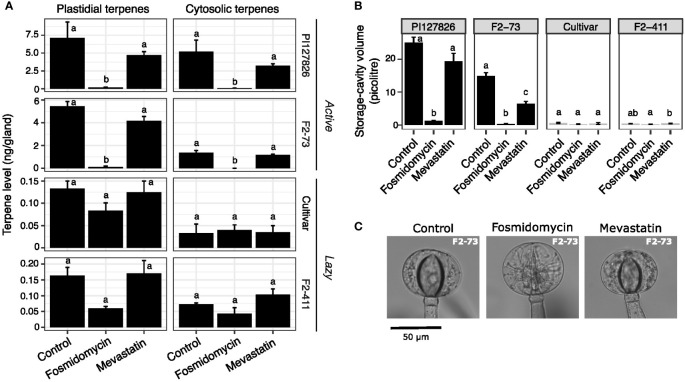
The effect of MEP/MVA pathway inhibition on plastidial-and cytosolically produced terpenes and the storage cavities. Cuttings (n = 3) of active (PI127826 and F2-73) and lazy (cultivar and F2-411) genotypes were grown for 14 days in hydroponic solution supplied with 10 μM fosmidomycin or 10 μM mevastatin. **(A)** Terpene levels in the type-VI trichome glands after 14 days of treatment. Terpene levels are summed according to the sub-cellular localisation (i.e. plastidial of cytosolic) of the corresponding terpene synthase (Zhou et al., 2020). The summed terpene quantities were Log_2_-transformed prior to ANOVA. Letters indicate significant groups (p < 0.05) after a Tukey HSD *post-hoc* test. **(B)** Volume of the storage cavity after 14 days of treatment. Letters indicate significant groups (p < 0.05) according to Tukey HSD *post-hoc* test after ANOVA of Log_2_-transformed cavity volumes **(C)** Representative microscope images type-VI trichome glands on the leaves of F2-73 after 14 days of treatment.

As observed above, fosmidomycin treatment greatly reduced both plastid- and cytosol-derived terpenes in the glands of active genotypes (**Figure 5A**). In this experiment, the levels of plastid-derived 7epiZ and its derivatives were for 98% reduced in PI127826 glands. Gland cells of F2-73 also displayed a reduction of 98%; from 5.5 ng/gland in control conditions to 0.12 ng/gland after treatment. It was furthermore confirmed that blocking of the MEP pathway reduces cytosolic terpenes (mainly β-caryophyllene, α-humulene and germacrenes) as well. Those terpenes were for 99% depleted in PI127826 (5.2 ng/gland in control conditions versus 0.065 ng/gland upon treatment) and were completely absent in glands of F2-73 (from 1.4 ng/gland control conditions versus undetectable levels upon treatent). In comparison to fosmidomycin, the effect of mevastatin on terpenoid production in “active” glands was less pronounced. Though both plastidial and cytosolic terpenes appeared less abundant upon inhibition of the MVA pathway, these differences were not significant (**Figure 5A**). Again, and especially apparent in the active genotypes, changes in terpene levels coincided with a reduction in storage-cavity volume, particularly apparent in active F2-73 (**Figures 5B, C**). This F2 exhibited a 60% reduction in volume under mevastatin-treatment compared to control (**Figure 5B**) indicating that also other, MVA-pathway dependent, products that were not analysed here, are present in the storage cavity.

Terpene levels of “lazy” genotypes F2-411 and the cultivar, were overall less affected by inhibitor treatment. In general, their trichomes contained much lower terpene levels compared to the active genotypes. Fosmidomycin reduced the levels of plastidial terpenes with 36% in the cultivar (from 0.13 to 0.083 ng/gland) and with 63% in F2-411 (0.16 to 0.060 ng/gland; **Figure 5A**). These reductions were however not statistically significant and the cavity volumes did not significantly alter under inhibition of the MEP pathway (**Figure 5B**). As for the “active” genotypes, no significant differences were observed in the abundance of either plastidial-or cytosolically synthesised terpenes of “lazy” genotypes treated with MVA-inhibitor mevastatin (**Figure 5A**).

### MEP pathway upregulated in gland secretory cells

**Figure 6:**
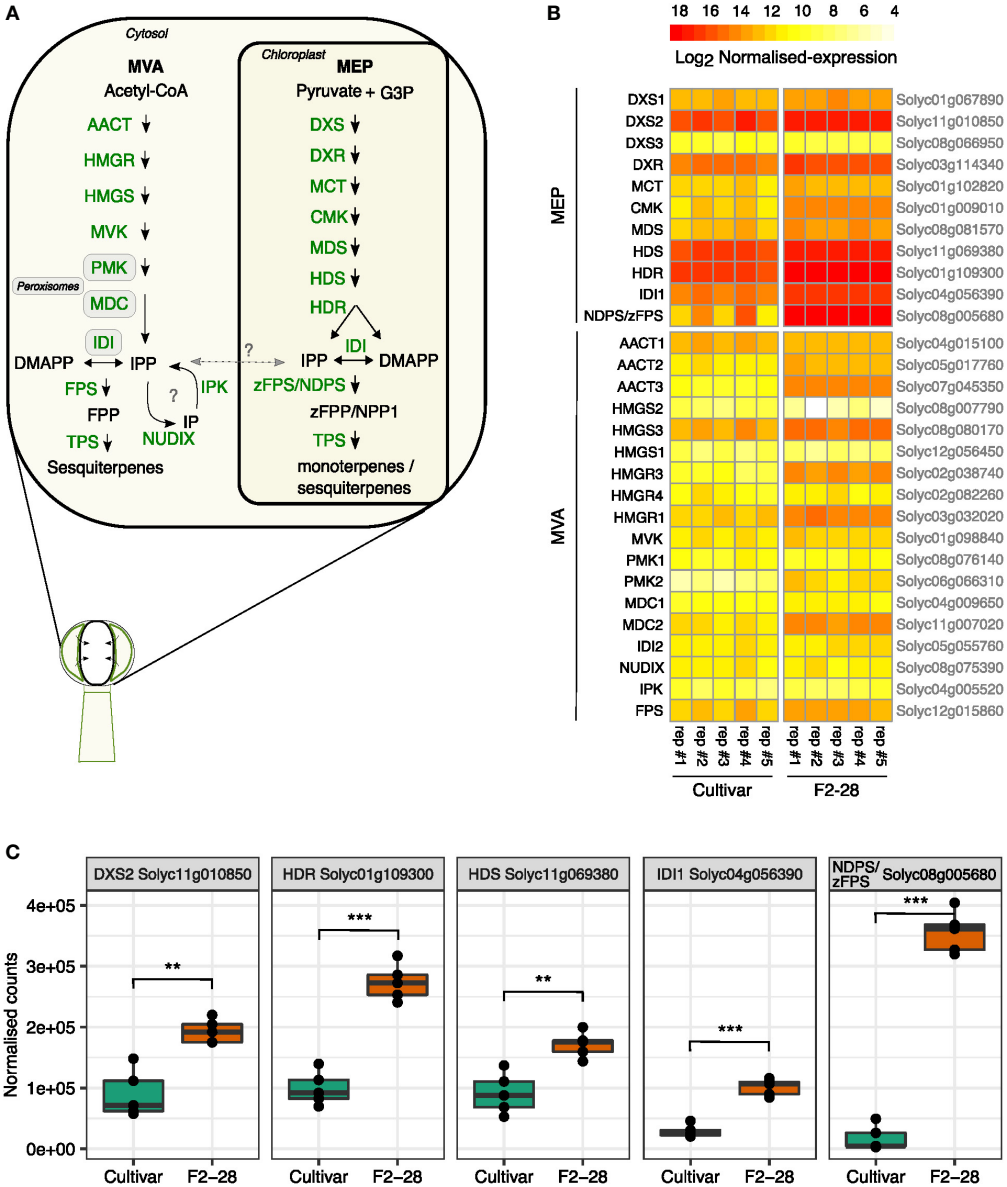
Gene expression patterns of the MEP and MVA pathway in secretory cells of F2-28 and the cultivar. **(A)** A schematic overview of the MEP and MVA pathway for mono-and sesquiterpene synthesis in the secretory cells of type-VI trichomes of tomato. Each arrow arrows indicates one enzymatic step. Metabolites are indicated in black, enzymes in green. Question marks indicate hypothetical steps that are not validated in tomato trichomes. **(B)** Heatmap showing the normalised counts (Log_2_ transformed) of transcripts obtained by mRNA-sequencing of secretory cells of type-VI trichomes isolated from the leaves of the cultivar (n = 5) and F2-28 (n = 5) cuttings. Plotted are transcripts mapping to the different homologous enzymes of the MEP and MVA pathway as encoded by genes in the *S. lycopersicum* genome (Heinz). Gene annotations are given on the left side of the heatmap, gene annotations are given on the right. **(C)** Boxplots of the normalised counts of the highest expressed genes in the MEP pathway of the cultivar and F2-28. Each dot represents one biological replicate. Asterisks show significant differences in expression (T-test) with ** p < 0.01 and *** p < 0.001. Enzyme abbreviations: 1-deoxy-d-xylulose 5-phosphate synthase (DXS); 1-deoxy-d-xylulose 5-phosphate reductoisomerase (DXR); 2-C-methyl-d-erythritol 4-phosphate cytidylyltransferase (MCT); 4-(cytidine 5′-diphospho)-2-C-methyl-D-erythritol kinase (CMK); 2-C-methyl-d-erythritol 2,4-cyclodiphosphate synthase (MDS); (*E*)-4-hydroxy-3-methylbut-2-enyl diphosphate synthase (HDS); (*E*)-4-hydroxy-3-methylbut-2-enyl diphosphate reductase; (HDR); isopentenyl diphosphate isomerase (IDI); *Z-Z*-farnesyl diphosphate synthase (zFPS); neryl diphosphate synthase (NDPS); terpene synthase (TPS); acetyl-CoA C-acetyltransferase (AACT); 3-hydroxy-3-methylglutaryl-CoA synthase (HMGS); 3-hydroxy-3-methylglutaryl-CoA reductase (HMGR); mevalonate kinase (MVK); phospho-mevalonate kinase (PMK); mevalonate-5-diphosphate decarboxylase (MDC); isopentenyl phosphate kinases (IPK); nudix hydrolase (NUDIX); farnesyl diphosphate synthase (FPS). Metabolite abbreviations: glyceraldehyde 3-phosphate (G3P); isopentenyl phosphate (IP); isoprene diphosphate (IPP); dimethylallyl diphosphate (DMAPP); neryl diphosphate (NPP); farnesyl diphosphate (FPP); *Z-Z*-farnesyl diphosphate (zFPP).

To investigate the transcript levels of the MVA- and MEP-pathway genes, we isolated the glands of type-VI trichomes from the leaves of the lazy cultivar and active F2 genotype F2-28 (**Figure 3B**) and compared gene expression by mRNA-sequencing of enzymes in the MVA and MEP precursor pathways (**Figure 6A**). The gland-isolation procedure involved a sieving step to enrich for intact type-VI glands resulting in only minimal contamination of other cells, or other trichome types (**Supplemental Figure S8**). We thus obtained mRNA profiles highly specific for the type-VI secretory cells.

In both genotypes, genes of the MEP pathway were relatively higher expressed than those of the MVA pathway (**Figure 6B**; **Supplemental Dataset 1**). Differential-expression analysis comparing the two genotypes showed that in the high-producing glands of F2-28, all genes of the MEP-pathway were significantly higher expressed compared to the cultivar (**Supplemental Figure S9**). The biggest difference in expression was found for genes in the last part of the MEP pathway; *zFPS* compared to *NDPS1* (20-fold), *IDI1* (4-fold), *HDR* (3-fold), *HDS* (2-fold) and the also first committed step of the pathway *DXS2* (2-fold; **Figure 6C**). Interestingly, most genes encoding enzymes of the MVA pathway were significantly higher expressed in F2-28 as well; *AACT3* (23-fold), *HMGS3* (3-fold), *HMGR3* (19-fold), *MVK* (1.5-fold), *PMK* (30-fold), *DMC1* (1.5-fold), *DMC2* (4.5-fold; **Supplemental Figure S9**; **Supplemental Dataset 1**). However, compared to the MEP pathway, expression levels of the MVA pathway genes were generally much lower. Moreover, in contrast to the MEP pathway, the final enzymatic steps of MVA pathway, *IDI2* and *FPS*, were equally expressed in the active F2-28 and the lazy cultivar (**Supplemental Figure S9**). Also, IPK and NUDIX, exhibiting relatively low expression levels in tomato secretory cells anyway, did not differ (**Supplemental Dataset 1**). Together, the results show that in the secretory cells MEP pathway genes are expressed much higher than the MVA pathway. In addition, transcript levels of the final steps of the plastidial-precursor pathway are particularly high in the active F2 trichomes compared to the cultivar.

Finally, the transcript levels of confirmed regulators of terpene biosynthesis in tomato trichomes were compared (**Supplemental Figure S10**). The expression of transcription factor *SCL3*, recently identified to be a regulator of *DXS2* (Yang et al., 2021) was 2-fold higher in F2-28 compared to the cultivar, in line with the higher transcript levels of *DXS2*. There was no differential expression in other transcription factors of potential interest. *EOT1*, *MYC1* and *MYB75* (Spyropoulou et al., 2014; Xu et al., 2018; Gong et al., 2021) did not significantly differ between the two genotypes studied here.

## Discussion

### High-level 7-epizingiberene production is a multigenic trait

We discovered that introgression of a metabolic trait into a tomato cultivar is challenging as the capacity to produce high quantities of terpenes seems to be constrained in a cultivated tomato (i.e. *S. lycopersicum*). Despite the fact that cultivated tomato has the suitable micro-organ to produce terpenes, i.e. a glandular-trichome type-VI, we showed that in the offspring of an interspecific cross with a high-producing wild tomato, terpene levels were much lower than expected based on the gene dosage of biosynthetic enzymes. The synthesis of sesquiterpenes, including 7epiZ and its derivatives, and monoterpenes is (co-)dominant, hence the F1 hybrid is able produce 7epiZ, 9HZ, 9H10epoZ as well as monoterpenes. However, terpene levels in F1 plants were only 7% of that of PI127826, and not significantly higher than those in a cultivar (**Figure 1A**). In contrast, terpene levels in the F1 made from two *S. habrochaites* accessions (F1-hab; LA1777 x PI127826) were similar to PI127826. This indicates that the germplasms of PI127826 and LA1777 share the (genetic) factors necessary to accumulate high levels of terpenes in type-VI trichomes or, alternatively, lack inhibiting factors that may be present in *S. lycopersicum*.

The pre-selection of the F2 plants for homozygous alleles of the full 7epiZ pathway (i.e. *zFPS*/*ZIS*/*ShZO*) allowed us to study rate limiting factors for high production in the plastid without interference of monoterpene synthesis *via NDPS1*/*PHS1.* The segregation of 7epiZ levels in this F2 population, which were highly skewed to low levels as found in the cultivar and the F1 (**Figure 2A**), indicated a multigenic recessive nature of high terpene production by type-VI trichomes. Our results are in line with previous findings describing trichome-produced specialised metabolite levels in segregating material of different interspecific crosses (Zamir et al., 1984; Frelichowski and Juvik, 2001; Zhang et al., 2008; Ben-Israel et al., 2009; Ji et al., 2022). Interspecific F1-hybrids made with various other *S. habrochaites* accessions (i.e. LA1777, LA1363, LA0407 and PI126449) or *S. pennellii* (LA0716) also displayed a disproportionate low production of specialised metabolites. Moreover, the frequency distributions of metabolite levels in segregating populations were also highly skewed towards the low levels found in the F1s (Zamir et al., 1984; Frelichowski and Juvik, 2001; Zhang et al., 2008; Ben-Israel et al., 2009; Ji et al., 2022). Similar frequency distributions were observed when F1-hybrids were back crossed to the wild parent pointing out the involvement of recessive alleles (Zamir et al., 1984; Frelichowski and Juvik, 2001).

Frelichowski and Juvik (2005) calculated three recessive loci to regulate santalene and bergamotene levels in LA1777. Likewise, biosynthesis of the methyl ketone 2-tridecanone in *S*. *habrochaites* LA0704, was predicted to require three genes in addition to the two known methyl ketone synthases (Fery and Kennedy, 1987). The genetic models that fit the frequency distribution of high 7epiZ levels observed here also comprise either three recessive loci, or two recessive loci plus one or two dominant ones (**Table 1**). These loci are irrespective of the *zFPS/ZIS* locus on chromosome 8 (Solyc08g005680 and Solyc08g005670) and the *ShZO* locus on chromosome 1 (Solyc01g008670), and do not seem to be linked. Noticeably, the examples above entail type-VI trichome specialised metabolites that originate from different precursor pathways. The SCAs in LA1777 and 7epiZ (derivatives) in PI127826 are MEP-pathway derived, while the terpenoids in LA1363 originate from the cytosolic MVA-pathway, and methyl-ketones (e.g. 2-tridecanone and 2-undecanone) are derived from (plastidial) fatty-acid biosynthesis. The factors that determine “high-level metabolite production” must therefore be involved in facilitating the flux, or enhance the storage capacity, independent of a particular pathway.

### Trichome activity and density are two independent traits

The total quantity of terpenes on a leaf-surface area is determined by a combination of type-VI trichome-density, the metabolic activity of the individual trichomes and their capacity to store and retain the metabolites. While it is obvious that the presence of high numbers of glandular trichomes contributes to the levels a plant can produce, trichome density alone could not explain the total terpene levels produced by the studied F2 plants. We found individuals with high numbers of trichomes producing relatively low levels of terpenes, and vice versa (**Figure 2B**; **Supplemental Figure S3B**). Hence, density can contribute, but does not explain high 7epiZ levels at the leaf level by itself. Likewise, the work of Ben-Israel et al. (2009) on the production of methyl-ketones in type-VI trichomes, showed that trichome density had a minor effect on total levels found on the leaf. Interestingly, trichome shape and allelic variants of the biosynthetic enzymes correlated better to the methyl-ketone concentrations (Ben-Israel et al., 2009). In conclusion, the “productivity” phenotype of type-VI trichomes segregates independently from trichome density (**Figure 2**) and should therefore be regarded as a separate trait that, in combination with density, will determine the total level of metabolites produced on the leaf surface.

Type-VI trichome density, typically higher in PI127826, appease to be co-dominant as the F1 hybrid exhibits a density phenotype intermediate to both parents (**Figure 1B**). A similar pattern was observed for type-I/IV and non-glandular trichomes (**Supplemental Figure S1**), suggesting that regulatory control over trichome development (e.g. initiation and type-differentiation) in general is regulated by co-dominant factors. Several studies indeed discovered trichome initiation and development is under control of transcription-factor protein-complexes that require all elements of the complex to be present (Guevara-García et al., 2005; Kang et al., 2016; Gao et al., 2017; Xu et al., 2018).

### Type-VI storage cavity volume and shape are determined by activity of the secretory cells

The difference in morphology of type-VI trichome glandular heads between *S. habrochaites* and *S. lycopersicum* accessions was proposed earlier to be related to the metabolite quantities inside the trichome (Bergau et al., 2015). The formation of “globular” shaped trichomes may be due to the “inflated-balloon” effect, where expansion of the internal cavity pushes the four secretory cells, encapsulated by a cuticular sac, outwards creating a spherical shape (Ben-Israel et al., 2009). The expansion of the cavity is likely facilitated by loosening of the gland-cell’s cell wall at the side facing the storage cavity, a process that seems to be more pronounced in *S. habrochaites* type-VI trichomes compared to *S. lycopersicum* (Bergau et al., 2015). Loosening of the cell walls, e.g. by pectinases or expansins, may therefore be a prerequisite for cavity expansion. Still, the accumulation of terpenoids remains a determining factor. The inhibitor assays done here show that the cavity volume in newly developed trichomes is dependent on the ability to produce terpenoids (**Figure 5B**). This “filling” of the cavity must take place predominantly during the very early stages of trichome development, as matured trichomes exposed to fosmidomycin treatment were much less affected (**Supplemental Figure S6**). This indicates that the volume of the storage cavity is not a predetermined trait but rather the consequence of metabolite accumulation early in trichome development. The fact that we observed only minor variation in cavity volume of trichomes on an individual plant, whether it is an “active” or “lazy” genotype (**Figure 3B**, lower panel), suggests that the metabolic activity of all secretory cells is co-regulated.

### A high metabolic flux through the MEP-pathway for the productivity of both plastidial as cytosolically localised terpene synthases

Compartmentalised production of IPP seems an efficient way to accommodate increased terpenoid production (Aharoni et al., 2003; Wu et al., 2006; Dong et al., 2016). With genes of the MEP pathway highly expressed, trichomes accumulated high amounts of terpenes *via* both cytosolic- and plastid-localised synthases (**Figure 5A**; Zhou and Pichersky, 2020). Evidence for precursor-crosstalk (i.e IPP/DMAPP) between plastids and the cytosol has accumulated over the past years (Hemmerlin et al., 2003; Liao et al., 2016; Henry et al., 2018; Gutensohn et al., 2021). The substantial reduction in terpene content observed after fosmidomycin treatment of active genotypes (**Figure 5A**) suggests that type-VI trichomes have a high metabolic flux through the MEP pathway driving the synthesis of terpenes in the plastid, and also in the cytosol. This indicates that also cytosolic terpene synthases in *S. habrochaites* and active F2-genotypes largely rely on precursors derived from MEP pathway. The contribution of the MVA pathway to the total pool of IPP in the secretory cells indeed seems limited as no significant effect was observed on either mono- or sesquiterpenes levels under treatment with mevastatin (**Figure 5A**). Combining those results with our gene-expression data (**Figure 6**), we hypothesised that the metabolic flux of isoprenoid precursors IPP/DMAPP in trichomes of *S. habrochaites* predominantly runs through a highly active MEP pathway. Such directional flux was described previously for snapdragon flowers (Dudareva et al., 2005) where sesquiterpene biosynthesis in the cytosol appeared to rely largely on the IPP generated by the MEP pathway. A bidirectional precursor-flux was shown previously for tomato fruit (Gutensohn et al., 2021). However, the MVA pathway contributes to only little amounts of terpenes in tomato trichomes (**Figure 5A**).

Although we did not investigate this further, we cannot exclude that genetic factors from *S. lycopersicum* repress the synthesis of terpenes. These could include (post-)transcriptional repressors of (regulators of) terpene biosynthesis, metabolite transport and storage capacity, the availability of carbon and energy or the branching of available carbon to other pathways (e.g. flavonoids). We did not see large differences in gene expression of (confirmed) transcriptional regulators of terpene metabolism, except for a 2-fold higher transcript level of *MIXTA-like* and *SCL3* which is in line with enhanced activation of the MEP pathway in the active F2-genotype. Through quantitative trait locus (QTL) mapping, Bennewitz et al. (2018) indicated multiple loci of *S. habrochaites* LA1777 correlated to the particular “globular” shape of its type-VI trichomes. They found a QTL on chromosome 1 that shows strong correlation to the shape, with additional loci on chromosome 7 and chromosome 11. As they used a backcross population, these QTLs indicate dominant loci. Here we showed the connection between terpene accumulation, storage-cavity volume and trichome morphology; the loci proposed by Bennewitz et al. (2018) may thus contain genes involved in terpene biosynthesis additional to gland shape. The enhanced metabolic flux can be the result of multiple upstream or downstream processes including transcriptional activation and the capacity to transport and store end-products in the storage cavity.

## Methods

### Plant material

The F1 was generated by transferring pollen from *S. habrochaites* PI127826 onto the stamen of a *S. lycopersicum* cultivar obtained from Enza Zaden Enkhuizen after which F1 seed was harvested. Next, the F1 was selfed to generate the *S. lycopersicum* x *S. habrochaites* PI127826 F2 population. F2 individuals were preselected for the presence of Tomato Mosaic Virus resistance of the cultivar and the 7-epizingiberene synthase (*ZIS;* Solyc08g005640), *zFPS* (Solyc08g005640) and a P450 (Solyc01g008650) from PI127826, using SNP markers. F1-hab was generated by crossing *S. habrochaites* PI127826 (male) to *S. habrochaites* LA1777 (female). The F2s, F1s and prenatal plants were maintained as cuttings and grown in enclosed climate-controlled greenhouse compartments (22–25°C; 16/8h light/dark). Biological replicates originate by growing multiple cuttings from the sample mother plant. All experiments were performed on ~4 weeks old cuttings except when indicated differently. Unless indicated otherwise, the leaf material and type-VI trichomes originated from 4^th^ fully developed leaf from the shoot apex, of which the lateral leaflets after the leaf’s terminal leaflet were taken.

### Trichome phenotyping

#### Trichome density

Two leaf discs (*r* = 2 mm) were taken per leaf surface (i.e. abaxial/adaxial) and the total number of non-glandular trichomes, type-VI trichomes and type I-and VI trichomes on each leaf disc was counted by eye under a stereo microscope. Per trichome type, the number of trichomes was summed over the abaxial-and adaxial surface to obtain the total number of trichome per leaf-disc area. Mean densities over the two leaf discs were taken to determine the density per biological replicate (n = 3 cuttings).

#### Type-VI trichome-density classes

To assign the F2 genotypes to trichome-density classes, as displayed in **Supplemental Figure S3A**, two leaf discs (*r* = 2 mm) were taken from each genotype. One leaf disc was used to estimate the number of type-VI trichomes on the abaxial surface, the second leaf disc for the abaxial surface. Next, the adaxial and adaxial surface were summed giving the total number of trichomes per leaf disc. For the parental genotypes, the mean summed number of trichomes was taken after outlier analysis (n= 4-5 cuttings).

#### Microscopy and storage-cavity measurements

Strips of the peripheral ends were cut from leaflets and mounted on a microscope slide, immersed in a drop of water and covered by a cover slip. Trichome images were taken with a EVOS™ digital inverted microscope (www.thermofisher.com) under bright light using a 20X objective. The height (medial plane) and width (transverse plane) of the cavities were measured using ImageJ (https://imagej.nih.gov) and the cavity volume was calculated according to the formula for prolate spheroids: volume = (4/3)*a ^2^*b, were *a* is the radius of the cavity’s width and *b* the radius of its height.

### Volatile analysis

#### Metabolite extraction

Leaflets were first weighted and subsequently submerged in 1mL *n*-hexane spiked with 0.5 ng/µL benzyl acetate as internal standard. After briefly rocking for 5 seconds the hexane was transferred to a 1.5 mL Eppendorf Safe-Lock tube containing ~10 mg Na_2_SO_4_(s) and vortexed for 5 seconds. The tubes were spun down for 5 minutes at 12.000 rpm where after the hexane extract was transferred to a 2 mL GC-MS glass vial and stored under N_2_(g) at -20°C until GC-MS analysis. For type-VI trichome gland-cell analysis, per sample 150 trichomes were collected from the adaxial side of a leaflet using the tip of a stretched-out glass Pasteur-pipette (final radius ~80-100 µM) while under a stereomicroscope. Volatiles were extracted in Eppendorf Safe-Lock tubes containing 300 µL *n*-hexane spiked with 0.5 ng/µL benzyl acetate. During this procedure, the tip of the Pasteur pipette picks up the type-VI head-cells by capillary force after brief contact. The pipette was washed in the *n*-hexane with every 30 head-cells collected to prevent clogging of the tip. After collection, ~10 mg Na_2_SO_4_(s) was added to the tube followed by 5 seconds of vortexing. The tubes were spun down for 5 minutes at 12.000 rpm where after ~150 µL of the hexane extract was transferred to a 2 mL GC-MS glass vial containing an autosampler insert and stored under N_2_(g) at -20°C until GC-MS analysis.

#### Data acquisition

Gas chromatograph-mass spectrometry was performed using an Agilent 7890A gas chromatograph coupled to an Agilent 7200 mass spectrometer. Depending on the sample, 1 or 2 μL of sample was injected, heated to 275°C and separated on a HP-5MS column (30 m × 250 μm x 0.25 μm; Agilent) using helium as carrier gas (flow rate: 1 mL min^−1^). The column was heated to 40°C for 3 min, after which the temperature increased 15°C/min up to 250°C which was held for 5 min. Ionisation of the sample was done at 70 eV with an emission of 35 μA at 230°C and data was collected with rate of 5 scans/second within a mass range of 40 – 300 mu.

#### Data analysis

Chromatographic peaks were integrated on the spectral base peak using Masshunter Quantitative software (Agilent). Peak areas were corrected for the internal standard, sample dilution, injected sample volume and, depending on the experiment, normalised for fresh leaf weight or number of collected trichomes. Normalised peak areas were quantified using authentic standards if possible. β-caryophyllene was used for quantification of 7-epizingiberene and its derivatives, α/β-bergamotene and α-santalene. The 95% confidence interval of PI127826 levels (n = 4) was determined by the mean peak area ± 1.96*(standard deviation/√n).

### Inhibition assays

Freshly made cuttings were grown on Terra Aquatica (www.terraaquatica.com/) hydroponic solution containing TriPart-Grow/TriPart-Micro/Tripart-Bloom/water: 1/1/1/9997 (v/v/v/v) in a climate-controlled greenhouse-compartment (22–25°C, 16/8h light/dark). After 1 week, roots emerged and cuttings were transferred to fresh hydroponic solutions supplemented with 10 μM fosmidomycin (Sigma-Aldrich, Catalog number: F8682) or 10 μM mevastatin (Sigma-Aldrich, Catalog number: M2537) or without inhibitors. After 14 days, the first lateral leaflets after the terminal leaflet of the 2^nd^ or 3^rd^ leaf from the apex were taken for microscopy and volatile analysis of the trichome head-cells. To ensure effective inhibitor treatment, cuttings were checked for development-related appearances that are typical after treatment with these inhibitors. Fosmidomycin-treated cuttings displayed bleaching of the newly formed leaves, while mevastatin-treated cuttings showed a stunted-growth phenotype (**Supplemental Figure S7**).

### Gland isolation and RNA extraction

Approximately 15-20 leaflets were collected in 50 mL tubes and were fully submerged in 30 mL ice-cold 70% EtOH. Tubes were hand-shaken for 20 times followed by 10 seconds of vortexing, whereafter the leaflets were briefly checked under a stereo microscopy for remaining type-VI trichomes. When many trichomes were still attached to the leaflets the shaking/vortexing step was repeated. The leaflets were next removed, and the extract was sieved through a 70 µm nylon cell strainer (Corning^®^ product # 431751) and collected into a fresh 50 mL tube. A small fraction on the collected samples (~50 µL) was inspected under a microscope for the enrichment of type-VI glands (**Supplemental Figure S8**) and to assure minimal contamination. The tubes were centrifuged at 3.000g for 5 minutes at 4°C. The pellet was resuspended in 1 mL 70% EtOH and, for each RNA-extraction, two resuspended pellets were combined into a fresh 2 mL Eppendorf^®^ tube. The Eppendorf tubes were centrifuged at 13.000g for 5 minutes at 4°C whereafter the supernatant was removed, and the pellet was frozen in N_2_ (l). Steel beads were added to the tube and shaken for 60 seconds using a paint shaker, then frozen in N_2_ (l) and stored at -80°C until further processing. The grinded tissue was used as input material for RNA extraction using the NucleoSpin^®^ RNA XS kit (Macherey-Nagel product # 740902) following manufacturer’s instructions and was eluted in 25 μL water. To further purify the RNA samples, 5 μL 10M of LiCl was added and samples were left overnight at -20°C. Precipitated RNA was spun down at 13.000 rpm for 20 minutes at 4°C and subsequently washed twice with 500μL 70% EtOH and resuspended in 10 μL water.

### Library preparation and mRNA sequencing

Prior to library preparation, poly-A enrichment was done using the NEBNext^®^ Poly(A) mRNA Magnetic Isolation Module (New England BioLabs). RNA-sequencing libraries were generated using the NEBNext^®^ Ultra II Directional RNA Library Prep Kit for Illumina with the NEBNext^®^ Multiplex Oligos for Illumina^®^ (Unique Dual Index Primer Pairs) according to the manufacturers’ protocols for larger insert size libraries (New England BioLabs). The size distribution of the libraries with indexed adapters was assessed using a 2200 TapeStation System with Agilent D5000 ScreenTapes (Agilent Technologies) and libraries were quantified using a QuantStudio 3 Real-Time PCR System (Thermo Fisher Scientific) using the NEBNext^®^ Library Quant Kit for Illumina (New England BioLabs) according to the manufacturer’s instructions. Libraries were clustered and sequenced (2x 150 bp) on a NovaSeq 6000 Sequencing System (Illumina) using a NovaSeq 6000 S4 Reagent Kit v1.5 (300 cycles, Illumnia). The mRNA-Seq reads were analysed using a custom Snakemake (Mölder et al., 2021) pipeline coined “snakemake-rnaseq” that is based on fastp, Kallisto and Subread (Liao et al., 2014; Bray et al., 2016; Chen et al., 2018). Read counts were normalised using DESeq2 in R (Love et al., 2014).

## Data availability statement

The original contributions presented in the study are publicly available. This data can be found here: https://github.com/BleekerLab/genetic_requirements/releases/tag/v0.5.0.

## Author contributions

PB, RK and MG conceived the study. RK and PB wrote the manuscript. RK, AM, RT and SM performed the experiments. MG performed the bioinformatic analyses. RS and MH provided input for the experiments and revised the manuscript. All authors contributed to the article and approved the submitted version.
