# Supplementary material for: Genetic and physiological requirements for high-level sesquiterpene-production in tomato glandular trichomes
Source: Front Plant Sci. 2023 Mar 3;14:1139274. doi: 10.3389/fpls.2023.1139274 (PMC10020594; doi:10.3389/fpls.2023.1139274)
Supplement: Supplementary file 1 [file DataSheet_1.pdf]

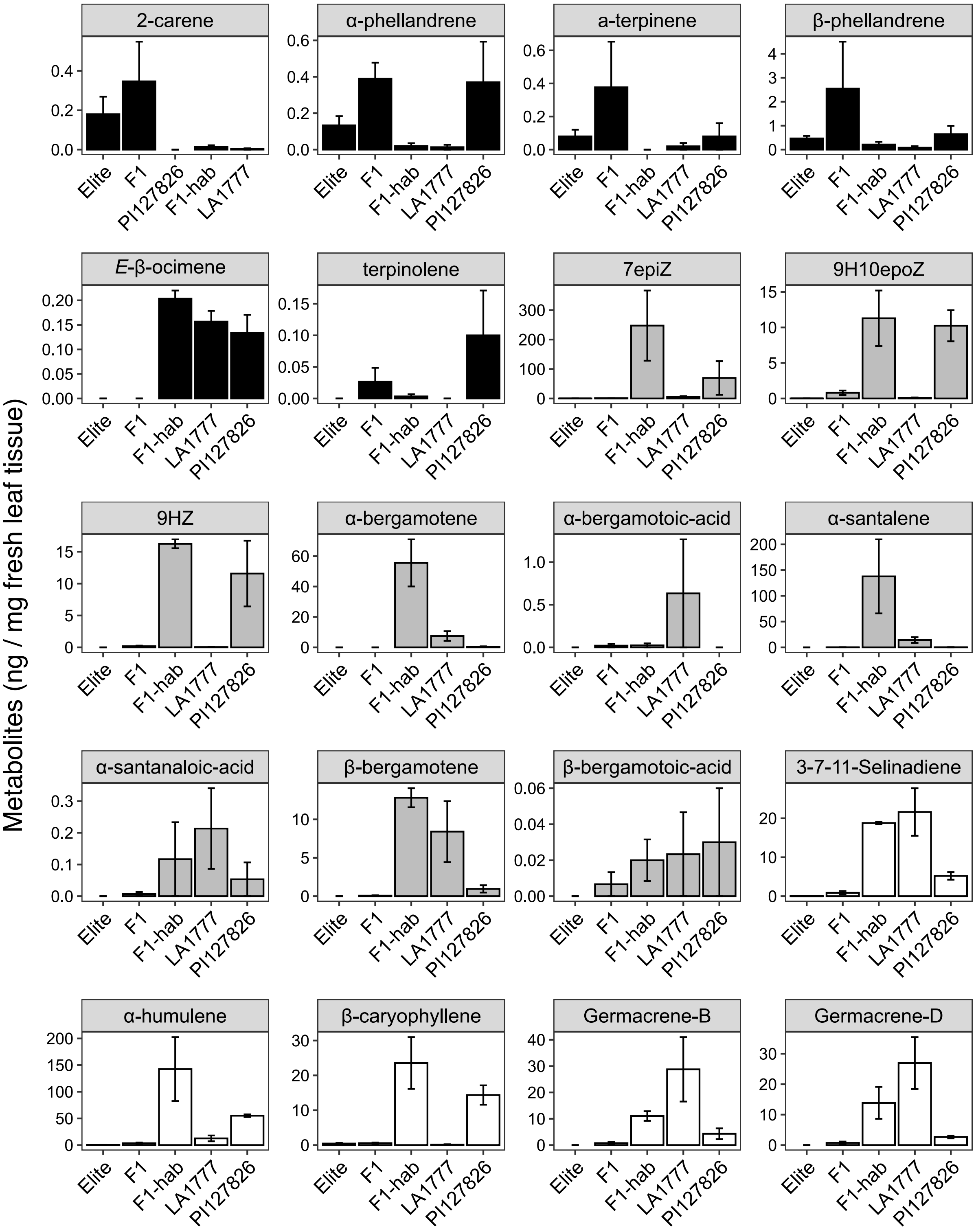

Supplemental Figure S1. Mono-and sesquiterpenes on the leaves of the tomato hybrids and their parents. Panels shows the individual terpenes detected on the leaves of the cultivar x PI127826 hybrid (F1), PI127826 x LA1777 hybrid (F1-hab) and their parents (n = 3). The colour of the bars indicates the type and cellular origin of the terpene: plastidial monoterpenes (black); plastidial sesquiterpenes (grey); cytosolic sesquiterpenes (white). Abbreviations used: 7epiZ: 7-epizingiberene; 9HZ: 9-hydroxy-zingiberene; 9H10epoZ: 9-hydroxy-10,11-epoxyzingiberene. The figure is supplementary to Figure 1A of the main text. The bars represent mean metabolite levels ± SE per mg fresh leaf weight.

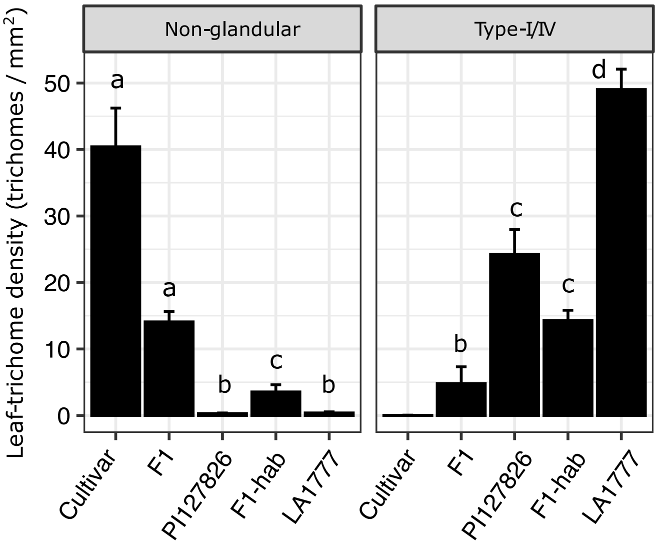

Supplemental Figure S2. Trichome densities on the leaves of the cultivar, PI127826, LA1777 and hybrids. The figure is supplementary to Figure 1B of the mean text and displays the non-glandular and type-I/IV trichome densities on the leaf. The black bars indicate the mean (n = 3) number of trichomes per mm2 SE. Statistically significant groups ($p < 0.05$) according to a Tukey HSD post-hoc test after ANOVA using log2-transformed densities are indicated by letters above the bars.

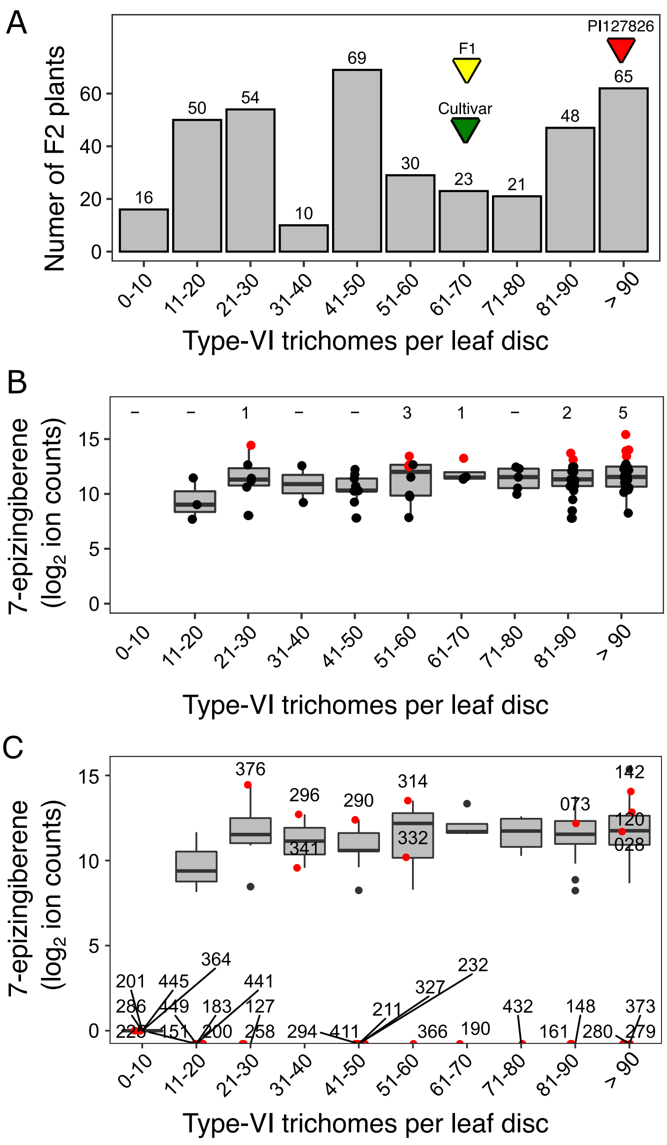

Supplemental Figure S3. Comparison of type-VI trichome densities and volatile levels on the leaf on a subpopulation of F2-plants. (A) The distribution of summed abaxial/adaxial trichome densities on leaflets of the F2 genotypes. The number in top of the bars indicate the number of F2-genotypes in each trichome-density class. Triangles indicate the average (n= 4-5) of the parental genotypes. **(B)** Log2-transformed ion counts of 7-epizingiberene per trichome-density class. Only F2s that produce 7-epizingiberene are plotted. Black dots indicate individual genotypes, red dots indicate genotypes with 7-epizingiberene levels comparable to PI127826 (95% confidence interval) or higher. The numbers on top of the boxplots indicate the number of red dots per trichome-density class. **(C)** Log2-transformed ion counts of 7-epizingiberene per trichome-density class as shown in B. By a red dot and their F2-identification number, this plot additionally indicates the F2-individuals that were selected to form the sub-population for detailed leaf-trichome density and terpene analysis (Figure 2B of the main text).

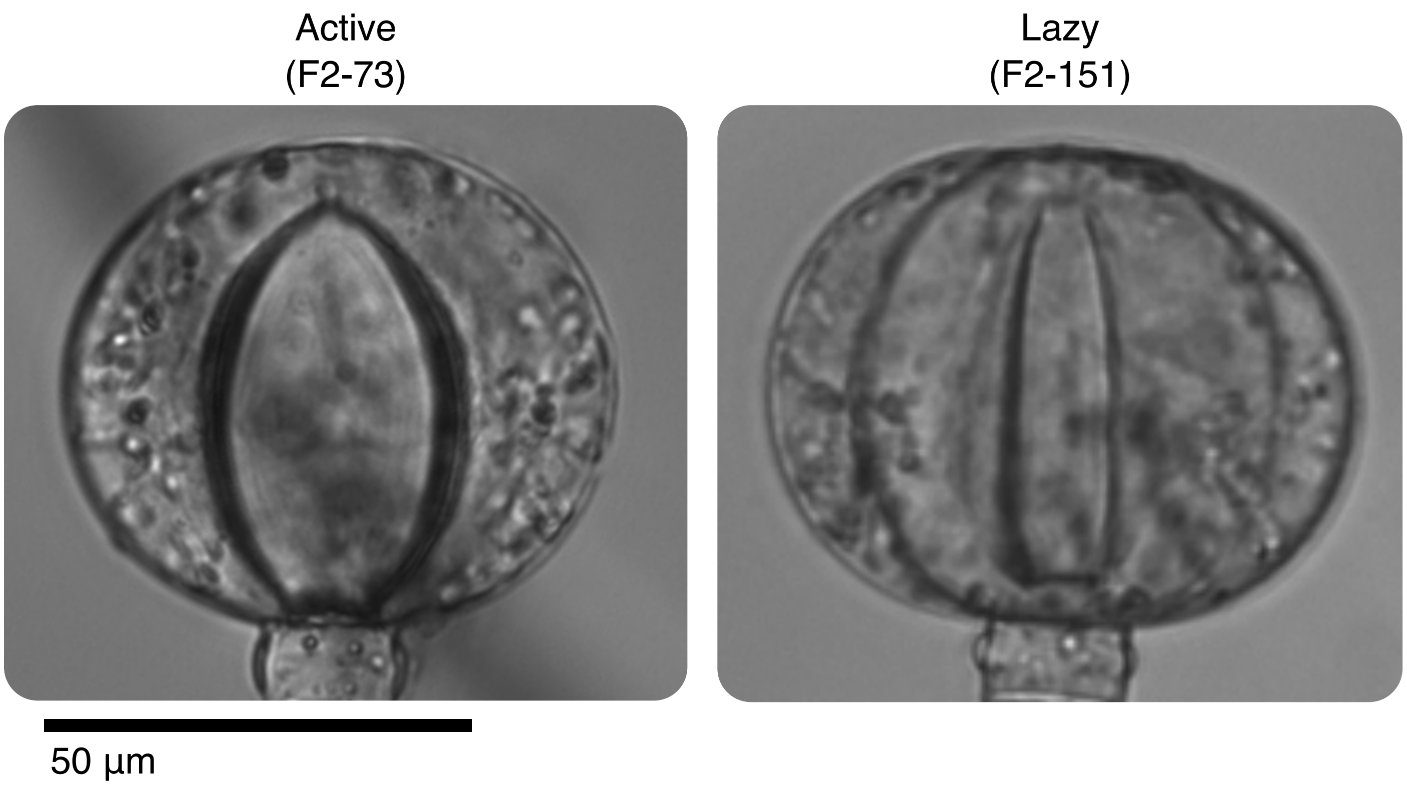

Supplemental Figure S4. Glandular heads of active and lazy type-VI trichomes. Enlarged photographs of the glandular heads of active (F2-73) and lazy (F2-151) F2 plants. The figure is supplementary to Figure 3 of the main text and demonstrates the difference in gland shape between active and lazy type-VI trichomes.

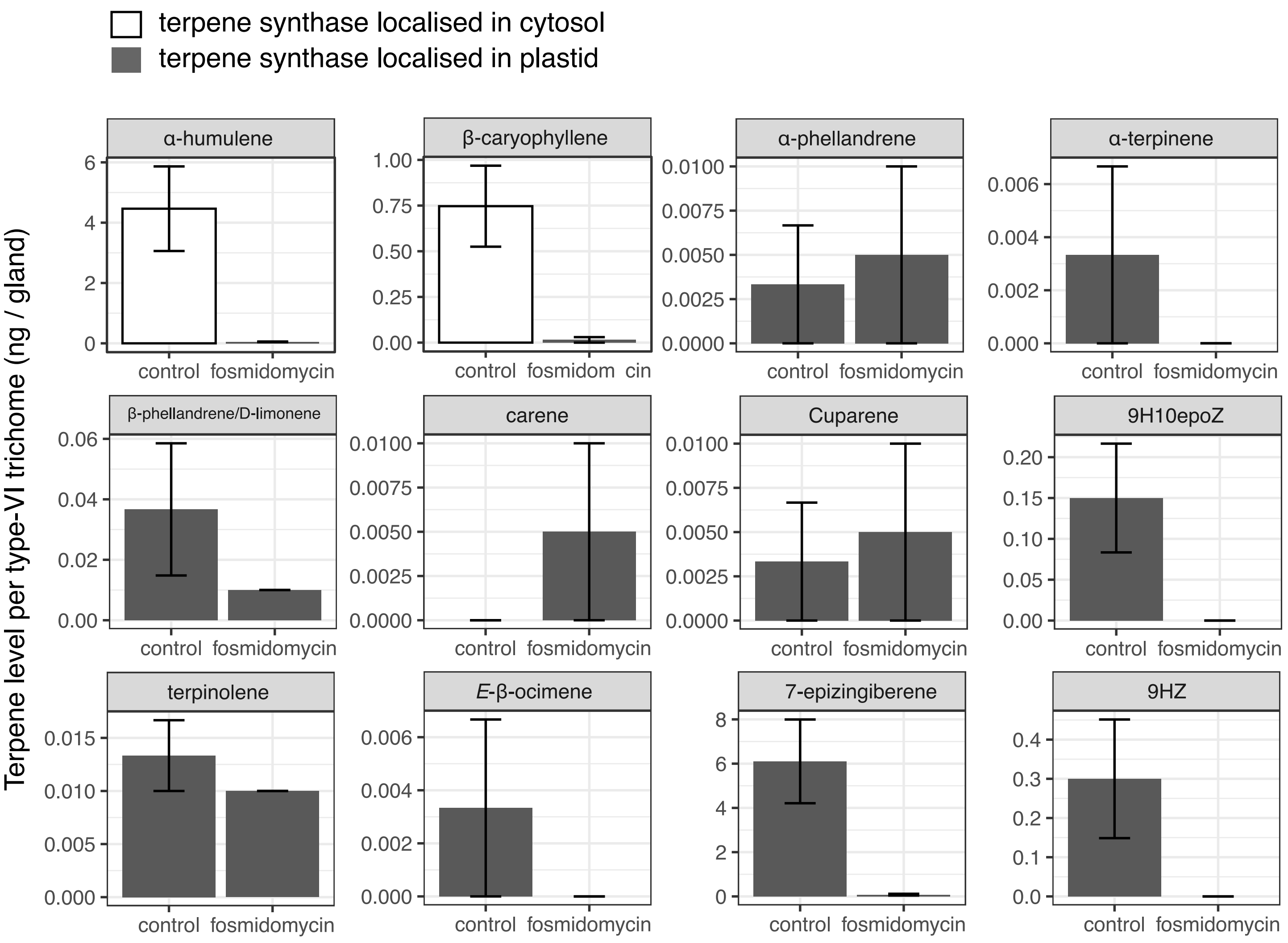

Supplemental Figure S5. Mono-and sesquiterpenes detected in the head cells of type-VI trichomes of PI127826 after fosmidomycin treatment. Bars represent the mean metabolite value in the head cells of the cuttings (n =3) SE. grown for 14 days in hydroponic solution supplied with or without 10 **μ**M fosmidomycin or without. Orange bars indicate terpenes originating from cytosolically localised terpene synthases; grey bars of plastid-localised terpene synthases according to Zhou et al. (2020). The figure is supplemental to Figure 4 of the main text and shows changes in levels of the individual terpenoids upon treatment.

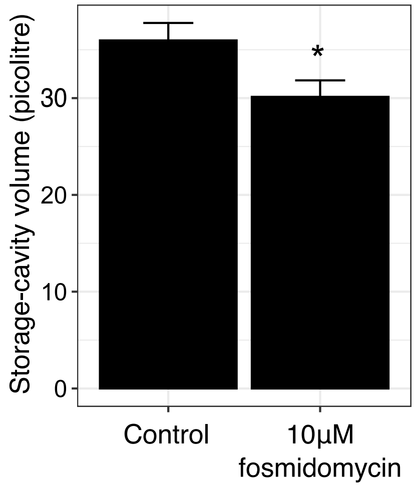

Supplemental Figure S6. Storage cavity volume of type-VI trichomes on leaflets formed prior to fosmidomycin treatment. The storage-cavity volume of type-VI trichomes that originate from leaflets that were already formed prior to the treatment. The measurements were done 14 days after treatment of PI127826 cuttings (n= 3) with 10µM fosmidomycin. The analysed trichomes. Bars represent de mean volume per cavity (n =50) SE. Asterisk shows the significant difference between the mean volume of the control and treated group (Mann–Whitney U test: $p < 0.05$).

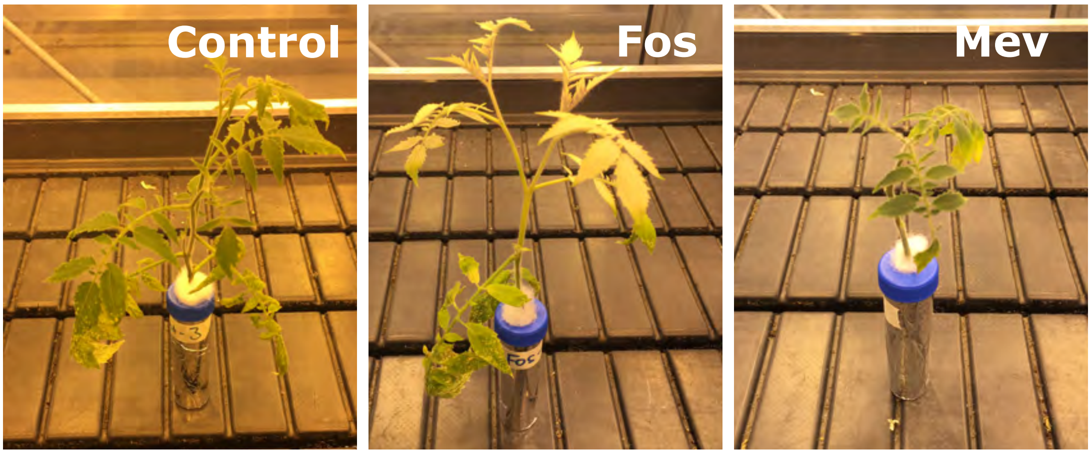

Supplemental Figure S7. Inhibitor treatments of the cuttings.
Photographs of PI127826 cuttings after growing for 14 days in hydroponic solution supplied with 10 µM fosmidomycin (Fos) or 10 µM mevastatin (Mev).

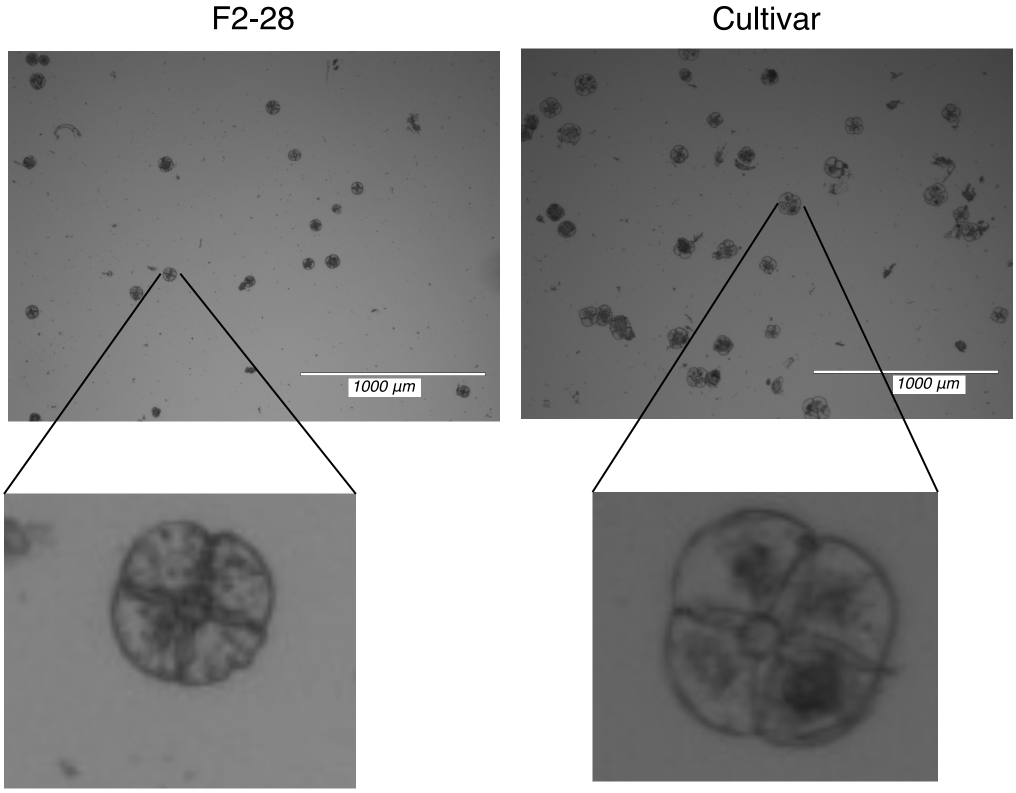

Supplemental Figure S8. Images of the isolation buffer after the gland-isolation procedure. Microscope images that are representative of the isolation-buffer after the last step of the gland isolation from leaves of F2-28 (left) and the cultivar (right) as was used as input for RNA extraction and subsequent mRNA-sequencing. The glands were still intact after the isolation procedure, with the four secretory cells in their typical formation as can be seen from the magnified part of the image.

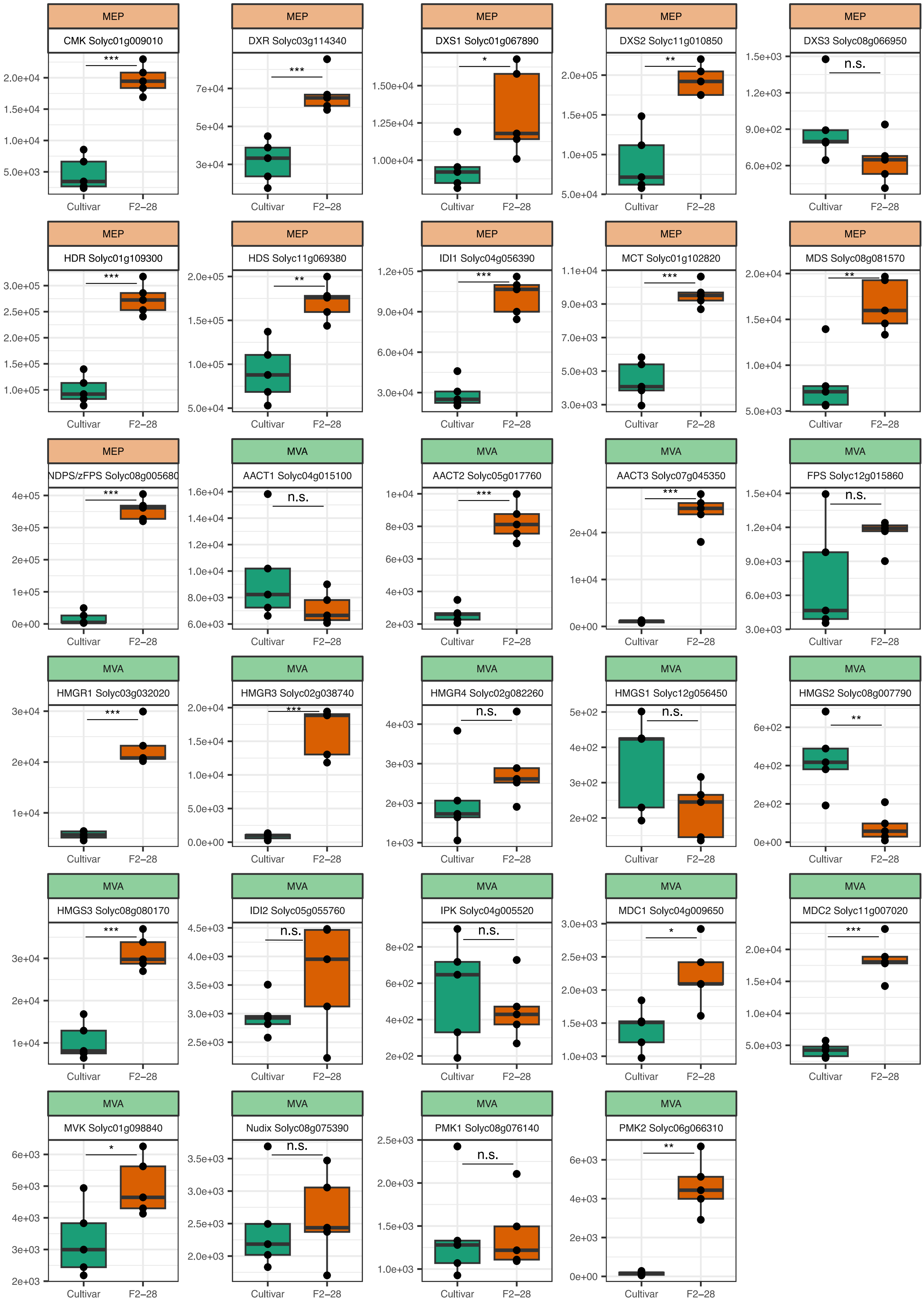

Supplemental Figure S9. Expression levels of genes in the MEP and MVA pathway.
The figure is supplemental to Figure 6 from the main text and shows the normalised counts of all MEP (orange strips) and MVA (green strips) genes as obtained by mRNA sequencing or the secretory cells of the cultivar (n = 5) and F2-28 (n = 5). The gene number and abbreviated annotation are given above the plots. Asterisks show significant differences in expression (T-test) with * $p < 0.05$; ** $p < 0.01$; *** $p < 0.001$; n.s: not significant. Note that the y-scale is differs per boxplot. The full list of raw and normalised counts for each replicate is available in Supplemental Dataset 1.

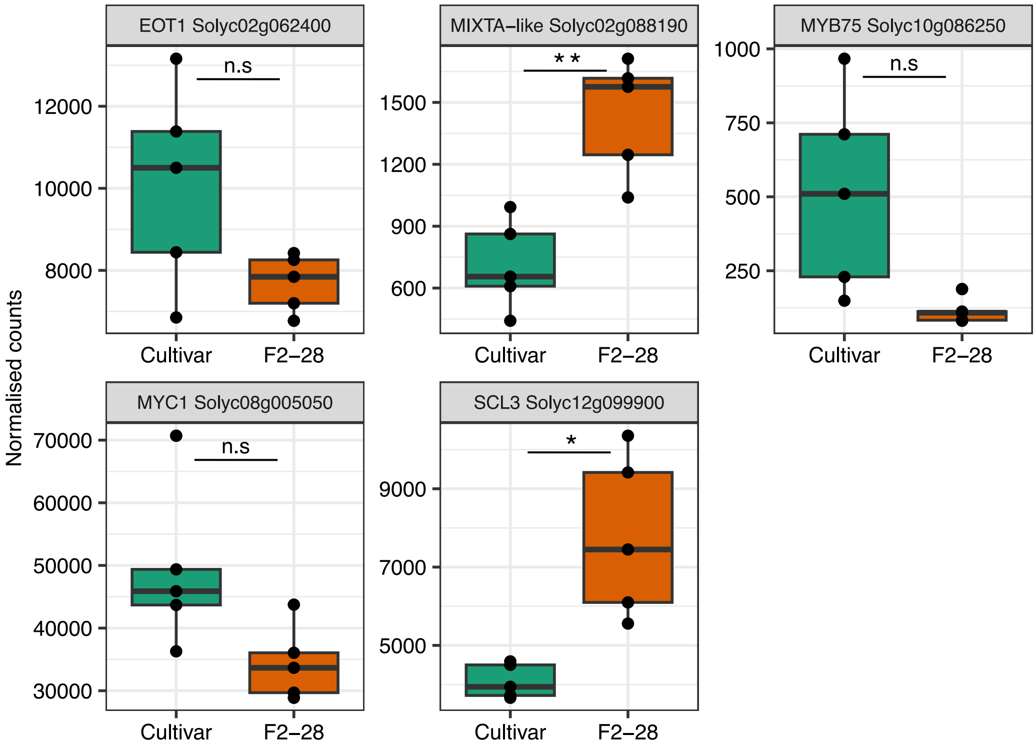

Supplemental Figure S10. Expression levels of confirmed regulators of terpene biosynthesis in tomato trichomes.

Boxplot show the normalised counts of transcription factors Emission of Terpenoids 1 (EOT1), MYC1 and Scarecrow-like 3 (SCL3) MIXTA-like and MYB75 obtained by mRNA sequencing or the secretory cells of the cultivar (n = 5) and F2-28 (n = 5). The dots indicate biological replicates. Asterisks show significant differences in expression (T-test) with * $p < 0.05$; n.s: not significant.
