# Supplementary material for: Genetic and physiological requirements for high-level sesquiterpene-production in tomato glandular trichomes
Source: Front Plant Sci. 2023 Mar 3;14:1139274. doi: 10.3389/fpls.2023.1139274 (PMC10020594; doi:10.3389/fpls.2023.1139274)
Supplement: Supplementary file 2 [file Table_1.docx]

**Supplemental Table S1.** Comparison of observed segregation of 7-epizingiberene plus derivatives to theoretical models

| Gene model | Segregation | χ^2^ | p-value |
| --- | --- | --- | --- |
| a - b | 1 : 15 | 3.93 | <0.05 |
| a - b - C | 1 : 20 | 0.75 | 0.38 |
| a - b - c | 1 : 63 | 13.0 | <0.01 |
| a - b - C - D | 1 : 27 | 0.07 | 0.79 |
| a - b - c - D | 1 : 84 | 23.7 | < 0.01 |
| a -b -c - d | 1 : 255 | 102 | < 0.01 |

Summing the peak areas of 7epiZ, 9HZ and 9H10epoZ results in 15 F2 plants having terpene levels of PI127826 of higher (95% confidence interval of higher). This 15:377 (1:25) segregation of high volatile levels was tested against expected segregation patterns of different genetic models as done in Table 1 of the main text. Gene models consist of recessive (small letters) and dominant (capital letters) loci. The theoretical segregation pattern of the phenotype expected under the corresponding gene model is given under Segregation. Models were compared to the observed 1:25 segregation using the Chi-square test for goodness-of-fit (χ^2^).
